# Value of Automatically Derived Full Thrombus Characteristics: An Explorative Study of Their Associations with Outcomes in Ischemic Stroke Patients

**DOI:** 10.3390/jcm13051388

**Published:** 2024-02-28

**Authors:** Mahsa Mojtahedi, Agnetha E. Bruggeman, Henk van Voorst, Elena Ponomareva, Manon Kappelhof, Aad van der Lugt, Jan W. Hoving, Bruna G. Dutra, Diederik Dippel, Fabiano Cavalcante, Lonneke Yo, Jonathan Coutinho, Josje Brouwer, Kilian Treurniet, Manon L. Tolhuisen, Natalie LeCouffe, Nerea Arrarte Terreros, Praneeta R. Konduri, Wim van Zwam, Yvo Roos, Charles B. L. M. Majoie, Bart J. Emmer, Henk A. Marquering

**Affiliations:** 1 Department of Biomedical Engineering and Physics, Amsterdam UMC, University of Amsterdam, 1105 AZ Amsterdam, The Netherlands; h.vanvoorst@amsterdamumc.nl (H.v.V.); m.l.tolhuisen@amsterdamumc.nl (M.L.T.); p.r.konduri@amsterdamumc.nl (P.R.K.); h.a.marquering@amsterdamumc.nl (H.A.M.); 2 Department of Radiology and Nuclear Medicine, Amsterdam University Medical Centers, 1105 AZ Amsterdam, The Netherlands; a.e.bruggeman@amsterdamumc.nl (A.E.B.); m.kappelhof@amsterdamumc.nl (M.K.); j.w.hoving@amsterdamumc.nl (J.W.H.); bruna.gdutra@gmail.com (B.G.D.); f.cavalcante@amsterdamumc.nl (F.C.); c.b.majoie@amsterdamumc.nl (C.B.L.M.M.); b.j.emmer@amsterdamumc.nl (B.J.E.); 3 Nicolab, 1105 BP Amsterdam, The Netherlands; eponomareva@nicolab.com; 4 Department of Radiology and Nuclear Medicine, Erasmus MC, 3015 GD Rotterdam, The Netherlands; a.vanderlugt@erasmusmc.nl; 5 Department of Neurology, Erasmus MC UMC, 3015 GD Rotterdam, The Netherlands; d.dippel@erasmusmc.nl; 6 Department of Radiology, Catharina Ziekenhuis, 5623 EJ Eindhoven, The Netherlands; 7 Department of Neurology, Amsterdam UMC, 1105 AZ Amsterdam, The Netherlands; j.coutinho@amsterdamumc.nl (J.C.); j.brouwer@amsterdamumc.nl (J.B.); y.b.roos@amsterdamumc.nl (Y.R.); 8 Research Bureau of Radiology and Nuclear Medicine, Amsterdam UMC, 1105 AZ Amsterdam, The Netherlands; k.m.treurniet@amsterdamumc.nl; 9 Department of Radiology, The Hague Medical Center, 2262 BA The Hague, The Netherlands; 10 Department of Radiology and Nuclear Medicine, Maastricht UMC, Cardiovascular Research Institute Maastricht (CARIM), 6229 HX Maastricht, The Netherlands; w.van.zwam@mumc.nl

**Keywords:** ischemic stroke, thrombus, artificial intelligence, imaging biomarkers, computed tomography scan

## Abstract

(1) **Background**: For acute ischemic strokes caused by large vessel occlusion, manually assessed thrombus volume and perviousness have been associated with treatment outcomes. However, the manual assessment of these characteristics is time-consuming and subject to inter-observer bias. Alternatively, a recently introduced fully automated deep learning-based algorithm can be used to consistently estimate full thrombus characteristics. Here, we exploratively assess the value of these novel biomarkers in terms of their association with stroke outcomes. (2) **Methods**: We studied two applications of automated full thrombus characterization as follows: one in a randomized trial, MR CLEAN-NO IV (*n* = 314), and another in a Dutch nationwide registry, MR CLEAN Registry (*n* = 1839). We used an automatic pipeline to determine the thrombus volume, perviousness, density, and heterogeneity. We assessed their relationship with the functional outcome defined as the modified Rankin Scale (mRS) at 90 days and two technical success measures as follows: successful final reperfusion, which is defined as an eTICI score of 2b-3, and successful first-pass reperfusion (FPS). (3) **Results**: Higher perviousness was significantly related to a better mRS in both MR CLEAN-NO IV and the MR CLEAN Registry. A lower thrombus volume and lower heterogeneity were only significantly related to better mRS scores in the MR CLEAN Registry. Only lower thrombus heterogeneity was significantly related to technical success; it was significantly related to a higher chance of FPS in the MR CLEAN-NO IV trial (OR = 0.55, 95% CI: 0.31–0.98) and successful reperfusion in the MR CLEAN Registry (OR = 0.88, 95% CI: 0.78–0.99). (4) **Conclusions**: Thrombus characteristics derived from automatic entire thrombus segmentations are significantly related to stroke outcomes.

## 1. Introduction

The effectiveness of endovascular treatment (EVT) was shown for patients with large vessel occlusion (LVO) strokes in 2015 [1,2], making EVT the standard treatment for LVO patients arriving at the hospital within 6 h after the stroke onset. However, new trials have shown that a wider group of patients can benefit from this treatment [3,4]. With the increasing demand, identifying factors that can predict the outcome of EVT is of higher interest.

Collecting extensive clinical information from ischemic stroke patients who present themselves to the hospital in the acute phase is difficult and sometimes unfeasible. Imaging characteristics, on the other hand, are more rapidly and consistently available and contain pertinent information about the patient’s present condition. In LVO stroke patients, certain radiological imaging characteristics of the thrombus on CT scans, such as length, volume, perviousness, and density, have been shown to be related to various outcome measures of stroke treatment [5,6,7,8,9,10]. However, thrombus location is the only thrombus imaging characteristic that is currently taken into account during the triage of ischemic stroke patients [11]. One reason for focusing only on this imaging characteristic and disregarding the others, apart from its established association with the outcome, is the fact that the thrombus location can be estimated without any need for annotation.

Quantifying most thrombus imaging characteristics requires annotating the thrombus. Thrombus annotations are commonly performed manually [12]. One disadvantage of manual annotations is that they are time consuming and therefore cannot be used in the clinical setting of stroke treatment. Another disadvantage of manual annotations is that they are subject to inter-observer variation [13].

Unlike manual methods, automatic methods can be used to create full thrombus annotations for a large dataset with minimal effort [14,15]. Additionally, provided the same model is used for segmentation, there will be no inter-observer variability in the automatic annotations. Therefore, they can potentially pave the way for involving thrombus characteristics in making treatment decisions in the clinic. Previously, we developed an automatic thrombus segmentation algorithm that had a good agreement with manual annotations [16]. However, the value of the automatic segmentations for extracting thrombus characteristics has not yet been established.

In this exploratory imaging biometrics study, we investigate the association of full thrombus characteristics calculated from the automatic segmentations with the functional outcome and technical success of endovascular treatment (EVT). Automated thrombus analysis is particularly valuable in large datasets such as registries and trials. However, differences in trial and registry populations (caused by strict inclusion criteria of trials) can affect the observed significant associations [17]. Furthermore, combining these populations may influence relationships between thrombus imaging and outcomes. Therefore, we study the value of fully automatically generated thrombus characteristics in the two distinct populations of LVO stroke patients treated with EVT.

## 2. Materials and Methods

### 2.1. Patient Selection

We included patients from two populations as follows: the Multicenter Randomized Controlled Trial of Endovascular Treatment for Acute Ischemic Stroke in the Netherlands (MR CLEAN) Registry and from the MR CLEAN-NO IV trial. Studying these two populations allows us to evaluate the value of automated segmentations in two potentially relevant applications of automated thrombus segmentations as follows: large clinical trials and large registries.

The MR CLEAN Registry [18] was a prospective observational study that followed the completion of the MR CLEAN trial [1], which was the first trial demonstrating the benefit of endovascular treatment in LVO stroke patients. The registry enrolled all the patients who underwent EVT in the Netherlands between March 2014 and January 2019, with eligible patients receiving intravenous thrombolysis (IVT) before EVT.

MR CLEAN-NO IV [19] was a prospective trial where patients with LVO were randomized between treatment with IVT prior to EVT or EVT alone. This trial included patients from twenty centers in the Netherlands, France, and Belgium between 2018 and 2020. Only patients who were directly presented to an EVT-capable center within 4.5 h after the stroke onset were included. The baseline scans included in the NO IV trial were all acquired before IVT administration.

**Figure 1:**
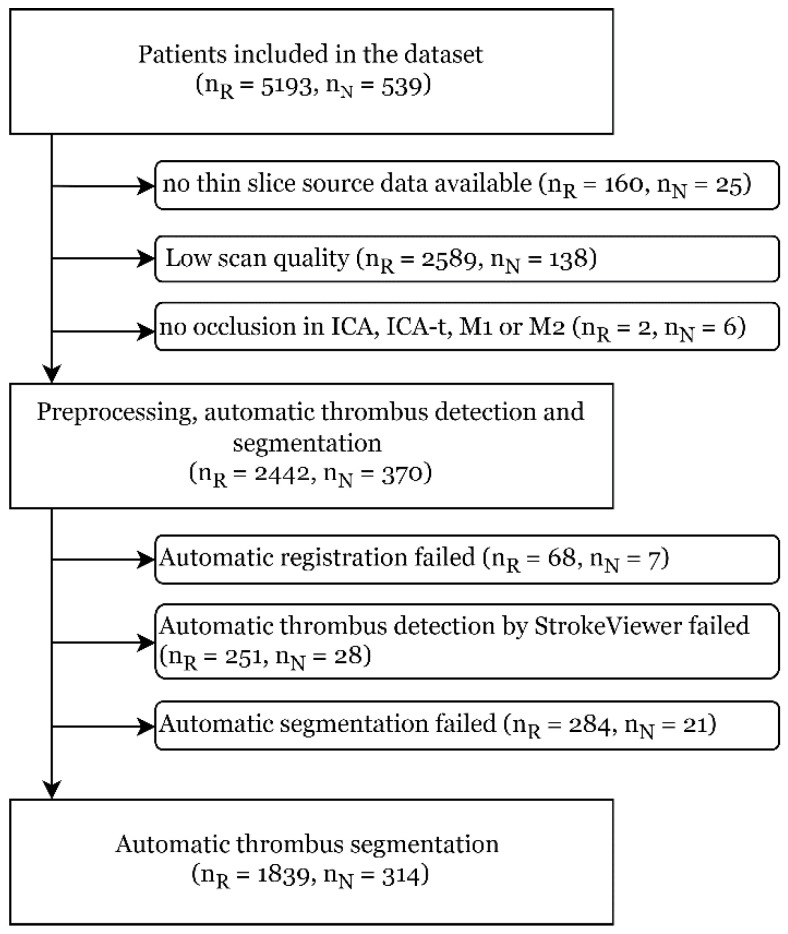
Flow chart of patient selection. $n_{R}$ refers to the number of patients in the Registry dataset, and $n_{N}$ refers to the number of patients in the NO IV dataset.

The patient selection procedure is schematically depicted in Figure 1. For each patient, the highest quality non-contrast CT (NCCT) and CT angiography (CTA) imaging available was used for further processing. Exclusion based on low scan quality includes a CTA slice thickness > 2 mm, NCCT slice thickness > 5 mm, number of slices < 8, the scan having incomplete brain coverage, the scan being made with sharp convolutional reconstruction kernels, or the scan having movement artefacts.

### 2.2. Automatic Thrombus Segmentation

We used a previously validated automatic thrombus segmentation algorithm [16]. The entire pipeline is automatic, and we used the output of the thrombus segmentation algorithm without any manual adjustments. The first step of the algorithm is to localize the thrombus with an off-the-shelf software, StrokeViewer LVO version 3.2.13 (Nicolab, Amsterdam, the Netherlands; www.nicolab.com/strokeviewer-home (accessed on 21 February 2024)). Subsequently, a neural network is used to segment the thrombus based on both the NCCT and CTA scans. The result is a 3D segmentation of the thrombus.

### 2.3. Thrombus Imaging Characteristics

**Figure 2:**
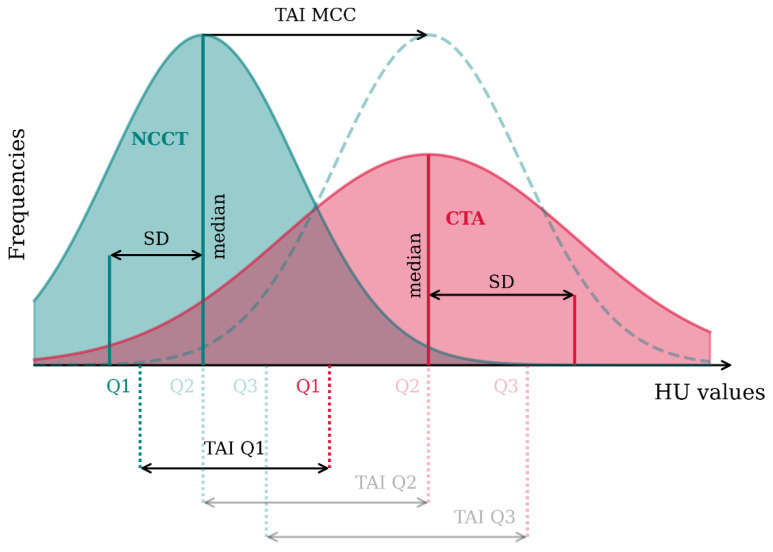
A representation of the thrombus characteristics that are calculated from the histogram of Hounsfield Unit (HU) intensity values of the thrombus in NCCT (green) and CTA (red). Three TAI (Thrombus Attenuation Increase) values are calculated from the difference between the first, second, and third quartiles (Q1, Q2, and Q3) of the two histograms. TAI_MCC_ (maximum cross correlation) is a measure of how much the NCCT histogram should be moved to achieve the maximum cross correlation with the CTA histogram. The distribution showed with a green, dashed line is the shifted NCCT histogram. Heterogeneity is defined as the CTA and NCCT SD (Standard Deviation). Density is shown as the CTA and NCCT median. In this figure, we have assumed the two histograms are normal distributions. In case of a non-normal distribution, median values do not overlap with the second quartile, and TAI_MCC_ is not necessarily the exact distance between the two medians.

We assessed the thrombus volume, perviousness, density, and heterogeneity. Thrombus perviousness quantifies thrombus permeability by comparing the average thrombus attenuation in the CTA and NCCT scans. This difference is named the Thrombus Attenuation Increase (TAI) [6]. Santos et al. (2021) proposed a method to calculate the TAI over the entire thrombus by comparing the intensity histograms of CTA and NCCT. This includes differences between the first (TAI_Q1_), second (TAI_Q2_), or third (TAI_Q3_) histogram quartiles, or the lag that results in the maximum cross correlation value between the two histograms (TAI_MCC_). Additionally, we included the median intensity of the thrombus in CTA as a measure of contrast penetration irrespective of thrombus density. Definitions of perviousness, density, and heterogeneity are depicted in Figure 2.

Thrombus density is the median density of the thrombus in NCCT (measured in HU values). We defined thrombus heterogeneity as the spread of density values in the thrombus and approximated it using the Standard Deviation (SD) of its histogram in both CTA and NCCT.

### 2.4. Outcome Measures

The association of the thrombus characteristics with the three outcome measures were assessed as follows: (1) the functional outcome measured on the modified Rankin Scale (mRS) after 90 days, (2) the successful final reperfusion defined as an expanded Thrombolysis in Cerebral Infarction (eTICI) score of 2b-3, and (3) the first-pass reperfusion success (FPS).

Ordinal mRS values were inverted as per previous MR CLEAN Registry studies [18], so odds ratios below one indicate a worse functional outcome. Successful reperfusion is defined as an eTICI score of 2b, 2c, or 3, indicating more than 50% reperfusion of the affected territory. FPS is defined as successful reperfusion after the first thrombectomy attempt.

### 2.5. Statistical Analysis

We report the baseline characteristics of the Registry and NO IV populations. Numerical variables were summarized using median and interquartile ranges, while categorical variables were presented as counts and percentages. The pre-stroke mRS and the Alberta Stroke Program Early CT Score (ASPECTS) were summarized by grouping some of their levels together. For the pre-stroke mRS, patients with an mRS of 2 or higher are grouped together. For the ASPECTS, three bins are made by grouping patients with an ASPECTS of 0 to 4, 5 to 7, and 8 to 10. In order to compare the two populations, Mann–Whitney U tests and Chi-square tests were used for numerical variables and categorical variables, respectively. Thrombus characteristics were summarized using the median and interquartile range and compared between populations using the Mann–Whitney U test. A *p*-value of <0.05 was considered to be significant.

We used logistic regression to investigate the associations between the thrombus characteristics and the outcome measures in both populations. Uni-variable ordinal and binary logistic regressions were used to study the relationship between the thrombus characteristics and the mRS, as well as the two technical success measures, respectively. Odds ratios (ORs) and 95% confidence intervals were reported to describe the effect of each thrombus characteristic on the outcome.

All statistical analyses were carried out using R (R version 4.0.5 (31 March 2021)). The missing values in the clinical data were imputed using multiple data imputation with additive regression, bootstrapping, and predictive mean matching using the Hmisc package (Frank E, 2021) with 5 imputations per missing value. Age, occlusion location, baseline NIHSS, administration of IVT, diabetes, onset-to-arterial puncture time, administration of the antiplatelet, atrial fibrillation, atherosclerosis, and collateral scores were used to inform the imputation. A sensitivity analysis was carried out for data imputation by undertaking a complete case analysis in which the cases with missing values were omitted and comparing the results with the imputed analysis (Appendix B).

We also performed multi-variable logistic regression analysis to investigate the effect of each thrombus characteristic on the selected outcome measures while making adjustments for the effect of the potential confounders (details are mentioned in Appendix A).

The consistency of the thrombus characteristics calculated from the automatic and manual segmentations was tested over a subset of the NO IV dataset in Appendix C.

## 3. Results

### 3.1. Population Characteristics

Table 1 summarizes the baseline characteristics of the two populations, showing significant differences between them. The median onset-to-groin time is more than twice as long in the Registry population compared with that of the NO-IV population. The percentage of patients who received IVT is considerably higher in the Registry population. Additionally, pre-stroke mRS scores ≥ 2 are more common for Registry patients. The collateral scores are better in the NO-IV population, and there are more M2 occlusions present in the Registry population than in the NO-IV one. The median age is higher in the Registry population, and there are more patients present with atherosclerosis in the NO-IV population. The time between the NCCT and CTA scans is longer in the Registry population.

First-pass success and successful reperfusion rates are higher in the NO-IV population. The mRS after a 90 day distribution is also significantly different between the two populations, where the percentage of patients with an mRS between 0 and 2 is 50% in the NO-IV population and 43% in the Registry one.

There are no missing data points for the mRS in the NO-IV population, but 9% of the data are missing for this outcome measure in the Registry one. The FPS data were missing for 25% in the Registry population and 12% in the NO-IV one. The missing data percentages for successful reperfusion were 4% for the Registry population and 9% for the NO-IV one.

The distributions of the thrombus characteristics in both the populations are presented in Table 2. Apart from the volume, the distribution of all the thrombus characteristics differed between the two population. Thrombi were more pervious and heterogeneous in the NO IV population than in the Registry one.

### 3.2. Associations with the Outcome

In Table 3, the univariate associations between the thrombus characteristics and the outcome measures are presented. For multiple thrombus characteristics, a statistically significant association with the outcome is observed.

In the Registry population, a lower volume, higher perviousness, and lower heterogeneity were significantly associated with a more favorable functional outcome. Perviousness was also significantly associated with the mRS in the NO IV population. However, volume and heterogeneity were neither statistically nor significantly related to the mRS in the NO IV population.

Only thrombus heterogeneity in NCCT was significantly related to FPS in the NO IV population, but none of the thrombus characteristics were significantly related to FPS in the Registry population. The effect of thrombus heterogeneity on FPS is large in the NO IV population where for every increase in heterogeneity (SD of NCCT) by 10 HUs, the chance of achieving first-pass success is reduced by 45%.

Thrombus heterogeneity in NCCT was the only thrombus characteristic that was significantly related to successful reperfusion in the Registry population where there is a higher chance to achieve successful reperfusion for less heterogeneous (more homogenous) thrombi. None of the thrombus characteristics were significantly associated with successful reperfusion in the NO IV population.

## 4. Discussion

In our study, fully automatically calculated thrombus characteristics were significantly associated with functional and procedural reperfusion outcomes in patients with acute ischemic strokes who were treated with EVT. Despite the differences between the two populations, perviousness was positively associated with a better functional outcome in both the Registry and NO IV trial, indicating a robust link between this biomarker and the functional outcome. The thrombus volume and heterogeneity were significantly associated with a worse functional outcome in the Registry population but not in the NO IV one. While the relationship between the volume and 90-day mRS is not significant in the NO IV population, we want to point out that the effect size in both populations is the same. Therefore, a potential cause for this difference in significance may be the smaller size of the NO IV population.

In this study, heterogeneity was the only thrombus characteristic significantly associated with reperfusion success; it was significantly associated with a lower chance of FPS in the NO IV population and with a lower chance of final successful reperfusion in the Registry population. Furthermore, heterogeneity in NCCT and perviousness estimated as the median density in CTA remained statistically and significantly associated with successful reperfusion even after adjusting for confounders. However, it is important to acknowledge a discrepancy between the two populations regarding the significant associations observed between heterogeneity and the outcome. Therefore, additional investigations are required to discern the patient groups for which heterogeneity can be a valuable biomarker. Furthermore, in this study we used a basic and intuitive definition for heterogeneity, while more complex formulations may be more descriptive of this biomarker [20,21].

We found a significant association between the thrombus volume and functional outcome in the Registry population. This association was also reported by Baek et al. (2017) [22] and van Voorst et al. (2023) [9], but it was not found by Borst et al. (2017) [23]. Unlike Baek et al. (2017) [22], we did not find a significant association between the thrombus volume and successful reperfusion.

We did not find a significant association between the density and any of the outcome measures. Similar results were found by Dutra et al. (2019) [5] for a 90-day mRS, by Baek et al. (2017) [22] for FPS, as well as by Jagani et al. (2017) [24] for successful reperfusion. Most of the studies that did find a relation between the thrombus density and outcome were focusing on IVT treatment only [25,26], thereby indicating that unlike IVT, thrombus density does not have an important role in EVT treatment.

Perviousness was the only thrombus characteristic significantly related to the 90-day mRS in both populations. The same relationship was observed on part I of the MR CLEAN Registry [5] and MR CLEAN trial populations for both sample-based [6] and entire thrombus [27] perviousness. Although Kappelhof et al. (2021) [28] did not find a significant association between perviousness and the mRS in patients who were treated with EVT in the pooled data of seven trials, they did find a positive association in patients treated with IVT. Similarly to previous studies, we did not find a significant association between perviousness and FPS [29] or successful reperfusion [28].

Our study provides the grounds to better understand the relationship between automatic thrombus characteristics and the outcome. Having a robust and reproducible measure of full thrombus analysis, such as that presented in our study, could aid in the understanding of the contradictory findings in previous studies. Despite omitting the inter-observer bias through the automatic annotations, we found that the significant associations between thrombus characteristics and outcomes differed between the MR CLEAN Registry and MR CLEAN-NO IV populations. These differences in significant associations could be caused by the restricted size of the populations. However, with the largest study size up to date, we do not think that this is the main cause of this difference. It is likely that the dissimilarities between the two populations contributes to the differences in the found associations. In future research, these population dissimilarities could be further explored to explain the causes of the observed associations based on the findings of this study. This is a necessary next step before creating a prognostics model to be used in the clinic. However, studying causality is outside of the scope of this paper.

The use of the automatic segmentations also allowed for the inclusion of a high number of patients in this study where we report on the largest patient population analyzed to date. Furthermore, the data used in this study were international and multi-centered, improving the generalizability of our results. However, it has to be noted that almost half of the patients had to be excluded because of low image quality. This was mostly due to a requirement for thin-slice images. Many centers do not save the thin-slice scans to avoid high storage costs. However, if the thrombus segmentation algorithm is integrated in the clinical workflow, it is possible to apply the algorithm on the thin-slice images before deleting them, thereby significantly decreasing the number of omitted scans.

Another strength of this study is that the analysis was not limited to one population. The data of patients who were enrolled in the MR CLEAN-NO IV trial were prospectively collected, and most patients had high-quality scans available, while the Registry dataset contained more patients with comorbidities, low-quality scans, and missing data compared with the MR CLEAN-NO IV population. Additionally, the quality of EVT treatment may have improved over the course of collecting the Registry data and beyond its completion. On the other hand, the Registry provides a realistic representation of the LVO patients who are eligible for EVT in the Netherlands. The trial data, with its carefully selected patients, closely resemble the populations commonly used to study the relationship between biomarkers and the outcome. On the other hand, the Registry data, containing more patients and being more reflective of real-world scenarios, offer insights into a broader patient cohort. We chose to analyze these populations separately to capture their unique attributes and strengths. Additionally, due to the smaller size of the MR CLEAN-NO IV population, we opted to not merge it with the Registry dataset to preserve its valuable information.

A limitation of our study is that of the high number of analyses conducted, thereby increasing the risk of accidental findings. Since we followed an exploratory analysis approach, we did not make corrections for multiple testing. However, it has to be noted that even though we looked at multiple definitions for some characteristics, we have only addressed four thrombus characteristics and three outcome measures.

In this analysis, thrombi were fully automatically segmented without manual adjustments, potentially leading to suboptimal segmentations in certain cases, such as occlusions in the proximal ICA or M2. Pseudo-occlusions were not explicitly inspected. However, the high number of patients included in this study is expected to account for the potential bias caused by these outliers. Additionally, we made use of both NCCT and CTA to better utilize the different information that each modality provides. However, this means that the segmentation method is only applicable in cases where both NCCT and CTA are available.

Finding causality and studying the confounding factors were not the focus of this study. In some of the adjusted analyses, associations were no longer statistically significant, indicating collinearity between parameters. It is shown that thrombus characteristics can be significantly associated with other factors such as etiology [30] and the collateral score [31]. However, an extensive evaluation of these collinearities is beyond the scope of our exploratory biomarker study. Additionally, it is relevant to know the relationship between the thrombus characteristics and outcome regardless of the confounding factors because imaging characteristics can be easily calculated from CT scans while confounding factors may not be known or easily calculable.

Finally, data were not available for all the outcome measures over the entire study population. We have used imputation for the bias that is caused by the possible non-random absence of data. However, since imputed values can only be based on non-missing data, they are not free of bias. This is especially the case for FPS in the Registry where the percentage of missing data were high. We conducted a complete case analysis in Appendix B to assess the potential bias.

## 5. Conclusions

Fully automatically computed thrombus characteristics from the entire thrombus are significantly associated with the functional outcome and technical success in EVT-treated anterior circulation acute ischemic strokes. We observed different, significant associations in the Registry and NO IV trial populations, thereby suggesting that these relations are population-specific. Nonetheless, we found that a lower thrombus volume, higher perviousness, and lower heterogeneity were significantly associated with a better functional outcome, while lower heterogeneity was correlated with improved reperfusion. These findings support the use of automatically assessed radiological thrombus characteristics in stroke research and clinical practice.

## Figures and Tables

**Table 1:** Descriptive characteristics of the included populations. Median and interquartile ranges (IQRs) are used to describe the continuous variables. Frequencies and percentages are reported for the categorical variables. In cases where data were missing, the number of available data is shown as “(n = x)”. “**†**” indicates a significant difference between the two populations. NIHSS: National Institutes of Health Stroke Scale; NCCT: Non-contrast Computed Tomography; CTA: Computed Tomography Angiography; mRS: Modified Rankin Scale; ASPECTS: Alberta Stroke Program Early CT Score; ICA: Internal Carotid Artery; ICA-t: ICA terminus; IVT: Intravenous Thrombolysis; eTICI: expanded Treatment in Cerebral Infarction

	MR CLEAN Registry (n = 1839)	MR CLEAN-NO IV (n = 314)	*p*-Value
Age, median (year, IQR)	72 (63–81)	70 (63–78)	0.03 **^†^**
Sex (men), n (%)	1007 (54.8%)	191 (60.8%)	0.05
Baseline NIHSS, median (IQR)	16 (11–20); (n = 1822)	17 (11–20)	0.09
Onset-to-arterial puncture time, median (IQR)	190 (138–269); (n = 1789)	92 (68–141)	<0.0001 **^†^**
Atherosclerosis (yes), n (%)	216 (11.7%)	49 (16.2%); (n = 303)	0.04 **^†^**
Diabetes (yes), n (%)	286 (15.7%); (n = 1825)	46 (14.6%)	0.71
Hypertension, n (%)	912 (50.6%); (n = 1801)	152 (48.6%)	0.54
Time between NCCT and CTA (minute), median (IQR)	13.4 (7.7–34.7); (n = 1609)	9.3 (6.8–13.4); (n = 197)	<0.0001 **^†^**
Pre-stroke mRS	(n = 1786)	(n = 313)	0.003 **^†^**
0	1166 (65.3%)	215 (68.7%)	
1	261 (14.6%)	59 (18.8%)	
≥2	359 (20.1%)	39 (12.5%)	
ASPECTS	(n = 1829)		0.21
0–4	79 (4.3%)	11 (3.5%)	
5–7	360 (19.7%)	50 (15.9%)	
8–10	1390 (76%)	253 (80.6%)	
Collateral score	(n = 1802)	(n = 311)	0.004 **^†^**
Score 0 (absent collaterals)	96 (5.3%)	21 (6.8%)	
Score 1 (filling = 50% of occluded area)	712 (39.5%)	90 (28.9%)	
Score 2 (>50% but less than <100%)	701 (38.9%)	137 (44.1%)	
3 (100% of the occluded area)	293 (16.3%)	63 (20.3%)	
Thrombus location	(n = 1825)		0.002 **^†^**
ICA	72 (3.9%)	1 (3%)	
ICA-t	380 (20.8%)	78 (24.8%)	
M1	1115 (61.1%)	200 (63.7%)	
M2	258 (14.1%)	35 (11.1%)	
Antiplatelet use (yes), n (%)	549 (30.2%); (n = 1819)	104 (33.1%)	0.33
IVT (yes), n (%)	1251 (68.4%); (n = 1830)	166 (52.9%)	<0.0001 **^†^**
First-pass success (yes), n(%)	467 (34%); (n = 1373)	143 (51.8%); (n = 276)	<0.0001 **^†^**
eTICI 2b/2c/3	1156 (65.8%); (n = 1757)	227 (79.1%); (n = 287)	<0.0001 **^†^**
mRS 90 days	(n = 1673)		<0.0001 **^†^**
0	129 (7.7%)	11 (3.5%)	
1	294 (17.6%)	36 (11.5%)	
2	296 (17.7%)	110 (35.0%)	
3	219 (13.1%)	35 (11.1%)	
4	188 (11.2%)	29 (9.2%)	
5	90 (5.4%)	31 (9.9%)	
6	457(27.3%)	62 (19.7%)	

**Table 2:** Comparing the thrombus characteristics between the two populations. Median and interquartile ranges (IQRs) are used to describe the variables. “**†**” indicates a significant difference between the two populations. TAI: Thrombus Attenuation Increase; SD: Standard Deviation; HU: Hounsfield Unit; NCCT: Non-contrast Computed Tomography; CTA: Computed Tomography Angiography; MCC: Maximum Cross Correlation

		MR CLEAN Registry (n = 1839)	MR CLEAN-NO IV (n = 314)	*p*-Value
Volume (mm^3^), median (IQR)		128 (49–261)	132 (53–271)	0.46
Density (HU)	NCCT median median (IQR)	47.6 (44.8–50.8)	49.2 (45.8–53.1)	<0.0001 **^†^**
Perviousness (HU)	TAI_Q1_ median (IQR)	0.51 (−5.0–6.5)	3.5 (−2.8–8.1)	<0.0001 **^†^**
	TAI_Q2_ median (IQR)	5.8 (−0.3–13.0)	9.6 (2.9–16.0)	<0.0001 **^†^**
	TAI_Q3_ median (IQR)	11.9 (3.9–22.0)	15.7 (8.6–25.0)	<0.0001 **^†^**
	TAI_MCC_ median (IQR)	4.3 (−1.6–10.9)	7.3 (0.0–12.7)	<0.0001 **^†^**
	CTA median median (IQR)	54.0 (47.0–61.7)	58.0 (53.0–65.7)	<0.0001 **^†^**
Heterogeneity (HU)	CTA SD median (IQR)	21.2 (15.7–18.1)	24.0 (18.3–31.3)	<0.0001 **^†^**
	NCCT SD (HU)	8.6 (6.7–10.6)	9.1 (7.1–11.1)	0.01 **^†^**

**Table 3:** Odds ratios relating the thrombus characteristics to the outcome measures in the univariate analysis. Numbers in the parenthesis show a 95% CI. Functional outcome: the reversed mRS (OR < 1 indicates a worse outcome); FPS: first-pass success, eTICI 2b-3 with a single thrombectomy device pass; eTICI2b+: successful reperfusion, eTICI ≥ 2b at the end of the procedure; HUs: Hounsfield Units; TAI: Thrombus Attenuation Increase; SD: Standard Deviation; OR: odds ratio; CI: confidence interval; NCCT: Non-contrast Computed Tomography; CTA: Computed Tomography Angiography; MCC: Maximum Cross Correlation and “**†**” and the bold text indicate a statistically significant relationship.

		VOLUME	DENSITY	PERVIOUSNESS					HETEROGENEITY	
		*Volume* (per 0.1 mL)	*NCCT median* (per 10 HU)	*TAI_Q1_* (per 10 HU)	*TAI_Q2_* (per 10 HU)	*TAI_Q3_* (per 10 HU)	*TAI_MCC_* (per 10 HU)	*CTA median* (per 10 HU)	*CTA SD* (per 10 HU)	*NCCT SD* (per 10 HU)
Functional Outcome	Registry	**0.91 ^†^** (0.87–0.95)	0.98 (0.87–1.09)	**1.14 ^†^** (1.07–1.21)	**1.10 ^†^** (1.04–1.15)	**1.05 ^†^** (1.02–1.09)	**1.11 ^†^** (1.05–1.17)	**1.09 ^†^** (1.03–1.15)	**0.93 ^†^** (0.87–0.99)	**0.87 ^†^** (0.77–0.98)
	NO IV	0.91 (0.81–1.02)	1.15 (0.81–1.62)	**1.20 ^†^** (1.01–1.44)	**1.22 ^†^** (1.04–1.42)	**1.16 ^†^** (1.02–1.30)	**1.17 ^†^** (0.01–1.35)	**1.23 ^†^** (1.06–1.44)	1.14 (0.94–1.40)	0.76 (0.53–1.09)
FPS	Registry	0.94 (0.88–1.00)	0.93 (0.80–1.08)	1.07 (0.97–1.18)	1.04 (0.96–1.12)	1.03 (0.98–1.08)	1.05 (0.97–1.14)	1.02 (0.96–1.10)	1.01 (0.92–1.12)	0.97 (0.84–1.13)
	NO IV	0.92 (0.79–1.06)	0.70 (0.44–1.12)	1.01 (0.82–1.25)	1.07 (0.89–1.29)	1.11 (0.95–1.29)	1.05 (0.87–1.27)	1.00 (0.84–1.20)	1.06 (0.84–1.34)	**0.55 ^†^** (0.31–0.98)
eTICI2b+	Registry	1.01 (0.95–1.06)	0.93 (0.82–1.05)	0.96 (0.89–1.03)	0.97 (0.91–1.03)	0.98 (0.94–1.03)	0.98 (0.92–1.05)	0.95 (0.90–1.01)	0.96 (0.89–1.04)	**0.88 ^†^** (0.78–0.99)
	NO IV	0.99 (0.84–1.17)	1.20 (0.72–2.00)	0.94 (0.70–1.26)	0.94 (0.75–1.17)	0.98 (0.83–1.16)	1.00 (0.81–1.23)	0.97 (0.77–1.22)	1.03 (0.76–1.38)	0.75 (0.47–1.21)

## Data Availability

Data for this study are available upon reasonable request.

## Appendix A

The odds ratios relating thrombus characteristics to the outcome after adjusting for confounders can be seen in Table A1. We made adjustments for age, occlusion location, the baseline National Institutes of Health Stroke Scale (NIHSS), IVT, diabetes, onset-to-arterial puncture time, antiplatelet, atrial fibrillation, atherosclerosis, and collateral scores.

None of the thrombus characteristics are independently related to the functional outcome and FPS in either population. However, perviousness formulated as the CTA median intensity and heterogeneity in NCCT are significantly related to successful reperfusion in the Registry population.

**Table A1:** Odds ratios for the multi-variable (adjusted) analysis. Numbers in the parenthesis show a 95% CI. Functional outcome: reversed mRS (OR < 1 indicates a worse outcome); FPS: first-pass success; eTICI2b+: successful reperfusion; HUs: Hounsfield Units; TAI: Thrombus Attenuation Increase; SD: Standard Deviation; OR: odds ratio; CI: confidence interval; NCCT: Non-contrast Computed Tomography; CTA: Computed Tomography Angiography; MCC: Maximum Cross Correlation and “**†**” and the bold text indicate a statistically significant relationship.

		VOLUME	DENSITY	PERVIOUSNESS					HETEROGENEITY	
		*Volume* (per 0.1 mL)	*NCCT Median* (per 10 HU)	*TAI_Q1_* (per 10 HU)	*TAI_Q2_* (per 10 HU)	*TAI_Q3_* (per 10 HU)	*TAI_MCC_* (per 10 HU)	*CTA Median* (per 10 HU)	*CTA SD* (per 10 HU)	*NCCT SD* (per 10 HU)
Functional Outcome	Registry	0.98 (0.93–1.03)	0.94 (0.83–1.05)	1.03 (0.97–1.10)	1.02 (0.96–1.07)	1.01 (0.97–1.04)	1.03 (0.97–1.09)	1.00 (0.95–1.06)	0.95 (0.89–1.02)	0.92 (0.83–1.03)
	NO IV	1.02 (0.90–1.17)	0.84 (0.58–1.22)	1.18 (0.97–1.43)	1.16 (0.98–1.38)	1.09 (0.96–1.25)	1.13 (0.97–1.32)	1.12 (0.95–1.32)	1.16 (0.94–1.43)	0.83 (0.56–1.23)
FPS	Registry	0.97 (0.90–1.04)	0.97 (0.83–1.12)	1.05 (0.95–1.17)	1.03 (0.95–1.10)	1.02 (0.97–1.07)	1.04 (0.96–1.13)	1.02 (0.95–1.10)	1.01 (0.91–1.12)	0.99 (0.85–1.14)
	NO IV	0.95 (0.80–1.12)	0.69 (0.41–1.15)	1.02 (0.81–1.27)	1.05 (0.86–1.28)	1.09 (0.93–1.29)	1.03 (0.84–1.26)	0.99 (0.81–1.20)	1.06 (0.83–1.35)	0.58 (0.32–1.02)
eTICI2b+	Registry	1.01 (0.95–1.07)	0.91 (0.80–1.04)	0.94 (0.87–1.01)	0.96 (0.90–1.02)	0.98 (0.93–1.02)	0.97 (0.90–1.04)	**0.94 ^†^** (0.88–1.00)	0.96 (0.89–1.04)	**0.87 ^†^** (0.78–0.99)
	NO IV	0.97 (0.79–1.19)	1.07 (0.63–1.82)	0.94 (0.68–1.29)	0.93 (0.72–1.21)	0.96 (0.79–1.15)	1.00 (0.80–1.26)	0.95 (0.75–1.22)	1.04 (0.76–1.43)	0.83 (0.50–1.36)

## Appendix B

For each outcome measure, a complete case analysis was performed where the cases with missing data were excluded and the uni-variable logistic regression was performed without data imputation. The results are reported in Table A2. One notable difference is that even though the effects are not different, the volume and perviousness formulated as TAI_Q1_ and TAI_MCC_ are correlated with FPS in the Registry population in the complete case analysis. Additionally, the direction of the effect of the volume on successful reperfusion is reversed between the imputed and complete case analysis for the NO IV population.

**Table A2:** Odds ratios of the complete case uni-variable analysis for both the Registry and NO IV populations. Numbers in the parenthesis show a 95% CI. Functional outcome: reversed mRS (OR < 1 indicates a worse outcome); FPS: first-pass success; eTICI2b+ =: successful reperfusion; HUs: Hounsfield Units; TAI: Thrombus Attenuation Increase; SD: Standard Deviation; OR: odds ratio; CI: confidence interval; NCCT: Non-contrast Computed Tomography; CTA: Computed Tomography Angiography; MCC: Maximum Cross Correlation and “**†**” and the bold text indicate a statistically significant relationship.

		VOLUME	DENSITY	PERVIOUSNESS					HETEROGENEITY	
		*Volume* (per 0.1 mL)	*NCCT Median* (per 10 HU)	*TAI_Q1_* (per 10 HU)	*TAI_Q2_* (per 10 HU)	*TAI_Q3_* (per 10 HU)	*TAI_MCC_* (per 10 HU)	*CTA Median* (per 10 HU)	*CTA SD* (per 10 HU)	*NCCT SD* (per 10 HU)
Functional Outcome	Registry	**0.90 ^†^** (0.86–0.95)	0.96 (0.86–1.07)	**1.14 ^†^** (1.07–1.22)	**1.10 ^†^** (1.04–1.16)	**1.06 ^†^** (1.02–1.10)	**1.11 ^†^** (1.05–1.18)	**1.09 ^†^** (1.04–1.15)	**0.93 ^†^** (0.86–0.99)	**0.85 ^†^** (0.75–0.96)
	NO IV	0.91 (0.81–1.02)	1.15 (0.82–1.62)	**1.20 ^†^** (1.01–1.44)	**1.22 ^†^** (1.04–1.42)	**1.16 ^†^** (1.02–1.30)	**1.17 ^†^** (1.01–1.35)	**1.23 ^†^** (1.06–1.44)	1.14 (0.94–1.40)	0.76 (0.53–1.09)
FPS	Registry	**0.93 ^†^** (0.87–0.99)	0.96 (0.83–1.12)	**1.11 ^†^** (1.00–1.22)	1.06 (0.98–1.15)	1.02 (0.97–1.08)	**1.09 ^†^** (1.00–1.19)	1.05 (0.97–1.14)	1.03 (0.94–1.13)	0.96 (0.84–1.11)
	NO IV	0.91 (0.79–1.04)	0.68 (0.44–1.04)	1.02 (0.82–1.27)	1.09 (0.90–1.32)	1.13 (0.97–1.32)	1.09 (0.90–1.31)	1.01 (0.84–1.21)	1.03 (0.81–1.31)	**0.54 ^†^** (0.30–0.97)
eTICI2b+	Registry	1.01 (0.95–1.06)	0.93 (0.82–1.06)	0.96 (0.89–1.03)	0.96 (0.91–1.03)	0.98 (0.94–1.03)	0.98 (0.92–1.05)	0.95 (0.89–1.01)	0.96 (0.88–1.04)	**0.87 ^†^** (0.77–0.98)
	NO IV	1.05 (0.88–1.24)	1.15 (0.69–1.90)	0.93 (0.72–1.21)	0.95 (0.76–1.19)	0.97 (0.82–1.16)	0.99 (0.79–1.23)	0.98 (0.79–1.22)	1.01 (0.76–1.36)	0.76 (0.47–1.24)

## Appendix C

In order to test the consistency between the manually and automatically calculated thrombus characteristics, a set of randomly chosen cases from MR CLEAN-NO IV was both manually and automatically segmented. Initially, the same subset of 100 patients included in the test set of [16] was used, and subsequently 2 patients were excluded because the automatic method did not produce a segmentation. Thrombus characteristics were calculated for both the manual and automatic segmentations and two-way mixed effects, and the single rater intra-class correlation coefficient (ICC) was calculated between them for each of the thrombus characteristics. The results are reported in Table A3. According to the guidelines provided by [32], to interpret the ICC, the automatically calculated TAI_Q1_, TAI_Q2_, TAI_MCC_, CTA median, and NCCT SD have an excellent agreement; the volume, NCCT median, and TAI_Q3_ have a good agreement; and the CTA SD has a fair agreement with the manually calculated characteristics.

**Table A3:** Intra-class correlation coefficient (ICC) between the thrombus characteristics calculated from the automatic and manual segmentations on a subset of the MR CLEAN-NO IV population.

	VOLUME	DENSITY	PERVIOUSNESS					HETEROGENEITY	
	*Volume*	*NCCT Median*	*TAI_Q1_*	*TAI_Q2_*	*TAI_Q3_*	*TAI_MCC_*	*CTA Median*	*CTA SD*	*NCCT SD*
ICC	0.60	0.62	0.88	0.81	0.63	0.85	0.84	0.43	0.87
